# Eastern Medicine: From Nutritional Supplements to Cancer Research

**DOI:** 10.1155/2014/817126

**Published:** 2014-12-21

**Authors:** Dominic P. Lu, Yemeng Chen, Lixian Xu, Leo M. Lee

**Affiliations:** ^1^University of Pennsylvania, Philadelphia, PA 19104, USA; ^2^New York College of Traditional Chinese Medicine, Mineola, NY 11501, USA; ^3^Department of Anesthesiology, School of Stomatology, Fourth Military Medical University, Xi'an, Shaanxi 710032, China; ^4^National Cancer Institute, National Institutes of Health, SAIC-Frederick, Inc., Frederick, MD 21702, USA

Throughout human history, complementary and alternative medicine in the form of folk medicine has emerged and flourished in every civilization, tribe, and continent. Some forms evolved to become traditional medicine and some disappeared to be forgotten, while others have been labeled untraditional medicine and are now regarded as complementary and alternative medicine. In the past half century, an increasing number of patients and health care providers in the West have become dissatisfied with aspects of traditional Western medicine and have turned their attention to these branches of untraditional medicine. The term integrative or integrated medicine was born recently as the popularity grew in incorporating complementary medicine to reinforce gaps to better fulfill the purpose of traditional medicine.

Complementary and alternative medicine includes many branches such as herbal medicine which increasingly appears in the form of nutritional supplements to elude increasing governmental regulation as demand for these products grow. In an analysis by the International Trade Center that spanned 2010–2013, it was estimated that global medicinal plant production was $50 billion and is growing at a rate of almost 16% annually. The increasing use of various forms of traditional herbal medicine in combatting modern illnesses, particularly the dangerous side effects of pharmaceutical drugs, has proven to be valuable. However, the absence of proper warning labels concerning drug-herb interaction causes an alarming number of emergency clinic cases due to the unwanted consequences of some of these interactions.

Acupuncture is one of these aforementioned branches that has made major inroads into Western medicine. It, along with the increased interest and research in herbal medicine, is likely the most researched branch of alternative medicine in the West. Acupuncture has been recognized for its healing value by the National Institutes of Health in 1997. The subsequent creation of the National Institute of Complementary and Alternative Medicine within the NIH in the United States and the founding of European Congress of Integrative Medicine has promoted research into these various overlooked disciplines. Understanding the value and discovering the merits of each discipline using modern Western scientific methodology is integral in trying to incorporate desirable aspects into traditional medicine. This special issue reviewed and accepted merited articles ranging in topics from the current dilemma of Eastern medicine in the West to the problem of government oversight in the field of herbs that are frequently and misguidedly marketed as nutritional supplements. An article included in this special issue titled “*Impact of Chinese herbal medicine on American society and health care system: perspective and concern*” reflected these issues and concerns. Also included are articles highlighting research into ginkgo biloba and cancer-related herbal research.

Noting all these development, we turn our attention to a systematic way of doing traditional Chinese medicine research. All articles published in this special issue underscore the positive trend of returning to natural approaches for our health care and emphasize better treatment for all types of human sickness.

There were three research articles, in this special issue, on the mechanisms of immune system and apoptosis for the therapeutic studies of antitumor activities. Both in vitro herbal drugs possess an enormous potential for the cure of certain types of cancer diseases.

It is important to have well-designed pharmaceutical studies to help explain the millennia old theory of Chinese herbology and the mechanism, pharmacodynamics, pharmacokinetics, and pharmacognostics that elucidate the efficacy of Chinese herbs and unlock its century-old mysteries. The traditional clinical application of traditional Chinese medicine has been based on the characteristics of taste, flavor, channel entering, and actions of the herbs. This special issue includes an article entitled “*Anti-inflammatory effects of 81 Chinese herb extracts and their correlation with the characteristics of traditional Chinese medicine*” which suggests that herbs with pungent flavors be considered the drugs of choice due to their effective anti-inflammatory agents which can be evaluated by their effects on nitrogen oxide (NO) production and cell growth in LPS/IFN*γ*-costimulated murine macrophage RAW264.7 cells. This discovery could be used as one of the criteria to select different Chinese herbs for anti-inflammatory purposes. Also included in this special issue is an intensive study on the effect of Cryptotanshinone, extracted from the Chinese herb Dan Shen (Salvia miltiorrhiza Bung) on reversing the reproductive and metabolic disturbances in polycystic ovary syndrome (PCOS) in rats. This study and its analysis into the possible regulatory mechanism would validate the clinical efficacy of this particular herb for the treatment of PCOS patients.

The guest editors of this special issue hope that, through the articles accepted and published, we can bridge the gap between Western and Eastern medicines and bring them closer in order to further understand the human body and to promote the advancement of health care. Western cancer treatments such as radiation and chemotherapy have adverse effect. Eastern medicine could help mitigate those side effects by minimizing the symptoms and reducing dosage requirements when used in conjunction with current Western treatment and therapies. Immunological, enzymatic molecular biology-related research could benefit from studies of effective Eastern medicinal treatments. Studies of the ways in which Western mainstream medicine and technology can be integrated with traditional Eastern medicine are the focus of this special issue.

*Dominic P. Lu* *Dominic P. Lu* *Yemeng Chen* *Yemeng Chen* *Lixian Xu* *Lixian Xu* *Leo M. Lee* *Leo M. Lee*

